# Supplementary material for: Malignant extracellular vesicles carrying MMP1 mRNA facilitate peritoneal dissemination in ovarian cancer
Source: Nat Commun. 2017 Mar 6;8:14470. doi: 10.1038/ncomms14470 (PMC5343481; doi:10.1038/ncomms14470)
Supplement: Supplementary Information — Supplementary Figures and Supplementary Tables. [file ncomms14470-s1.pdf]

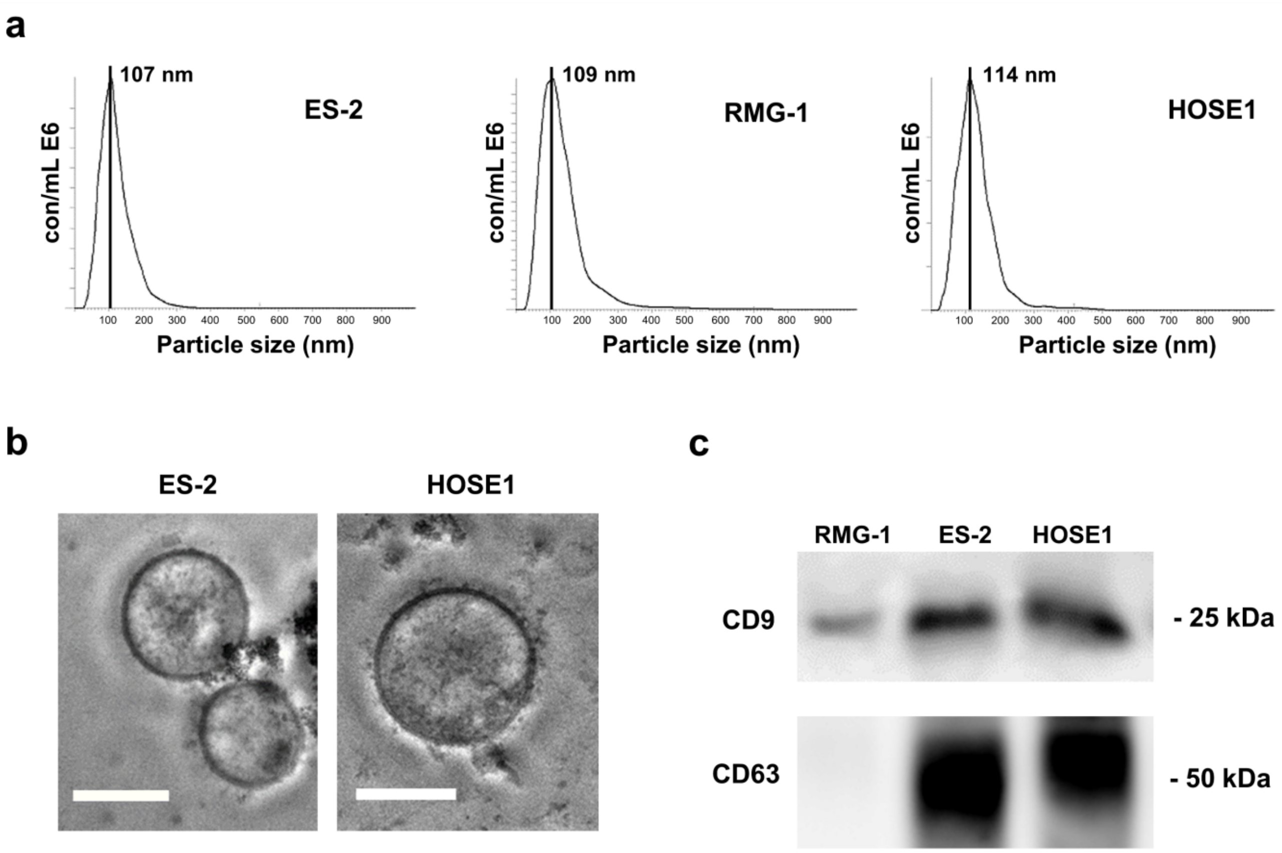

d

	ES-2	RMGI	HOSE1
**The number of total cells**	5.0.E+07	2.7.E+07	1.7.E+07
**Cell culture medium (ml)**	3.3.E+01	3.3.E+01	3.3.E+01
**Particle number / cell**	2.1.E+06	1.7.E+06	2.7.E+06
**RNA (pg) / cell**	4.3.E-01	4.5.E-02	7.1.E-02
**Protein (μg) / cell**	3.4.E-07	5.4.E-07	4.9.E-07
**Particle size (nm)**	107	109	117

Supplementary Figure 1. Characteristics of EVs (a) Nanoparticle tracking analyses of the particle size of ES-2 EVs, RMG-1 EVs and HOSE1 EVs. The vertical axis in the graphs shows the number of EV particles (x 10^6)/mL, and the horizontal axis indicates the particle size (nm). (b) Representative phase-contrast electron microscopic images of ES-2 EVs and HOSE1 EVs. Scale bar, 100 nm. (c) Immunoblot analysis of the conventional EV markers CD63 and CD9. EVs from ES-2, RMG-1 and HOSE1 cells were used. 50 ng / lane. (d) A table for EV characteristics. Comparisons of the particle number, the RNA concentration and protein concentration are shown.

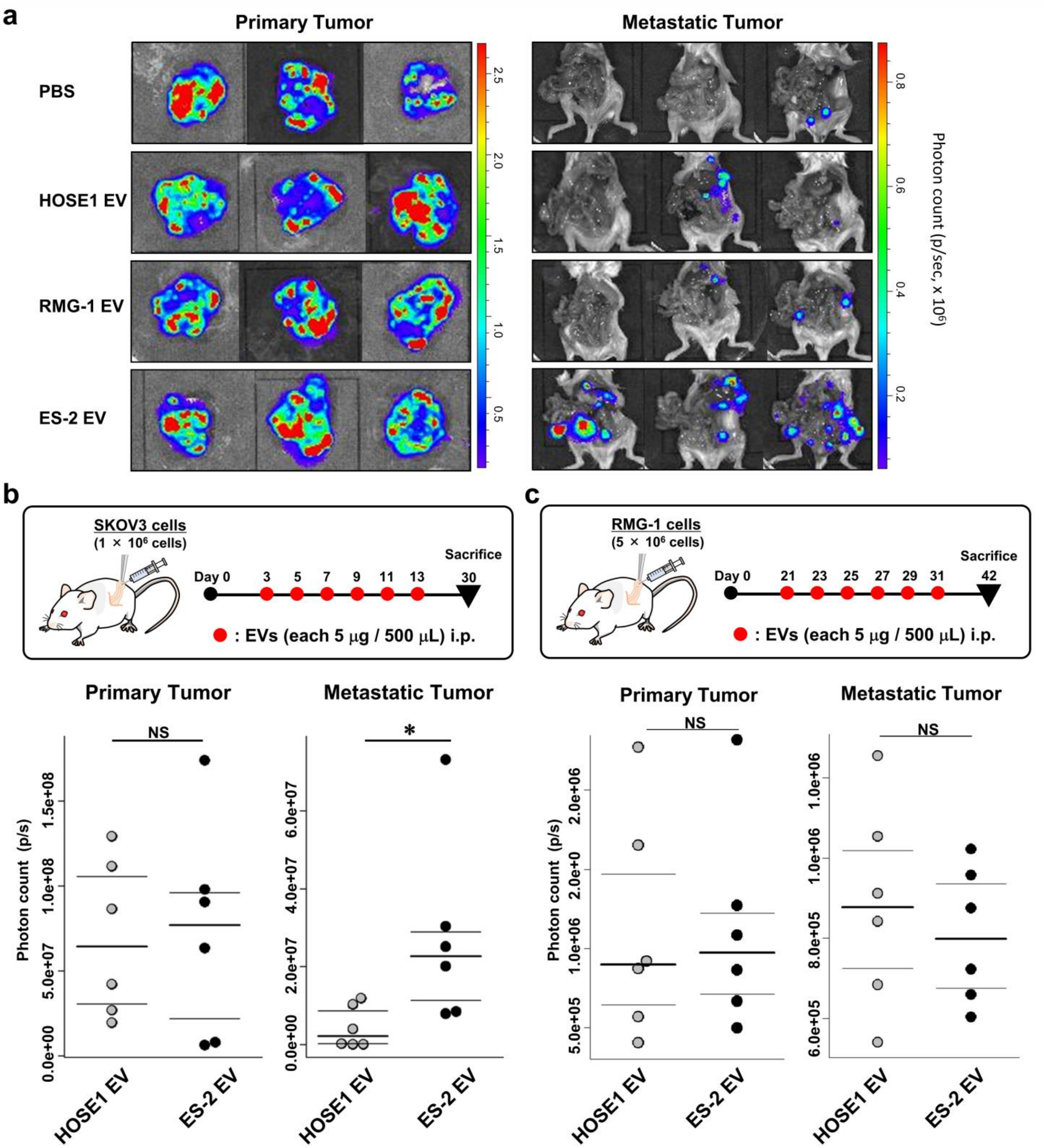

Supplementary Figure 2. In vivo experiments to determine whether EVs promote peritoneal dissemination of cancer cells (a) Representative bioluminescence images of the mice, comparing four types of EV treatment. Dissected primary tumors (left) and remaining metastatic tumors (right) are shown. (b) Upper: Schematic protocol for investigating the EVs in peritoneal dissemination. Orthotopic mouse model was established with SKOV3 cells. ES-2 EVs and HOSE1 EVs were injected i.p. from day 3 and every other day thereafter, for a total of 6 times. The mice were euthanized on day 30. Lower: Distribution of photon count in the dissected primary tumor (left) and the peritoneal metastatic tumors (right). n = 6; HOSE1 EV, n = 6; ES-2 EV. Student's t-test (**p* < 0.05 and NS = no significance). (c) Upper: Schematic protocol for investigating the EVs in peritoneal dissemination. Orthotopic mouse models were established with RMG-1 cells. ES-2 EVs and HOSE1 EVs were injected i.p. from day 21, every other day thereafter, for a total of 6 times. The mice were euthanized on day 42. Lower: Distribution of photon count in the dissected primary tumor (left) and the peritoneal metastatic tumors (right). n = 6; HOSE1 EV, n = 6; ES-2 EV. Student's t-test (**p* < 0.05 and NS = no significance).

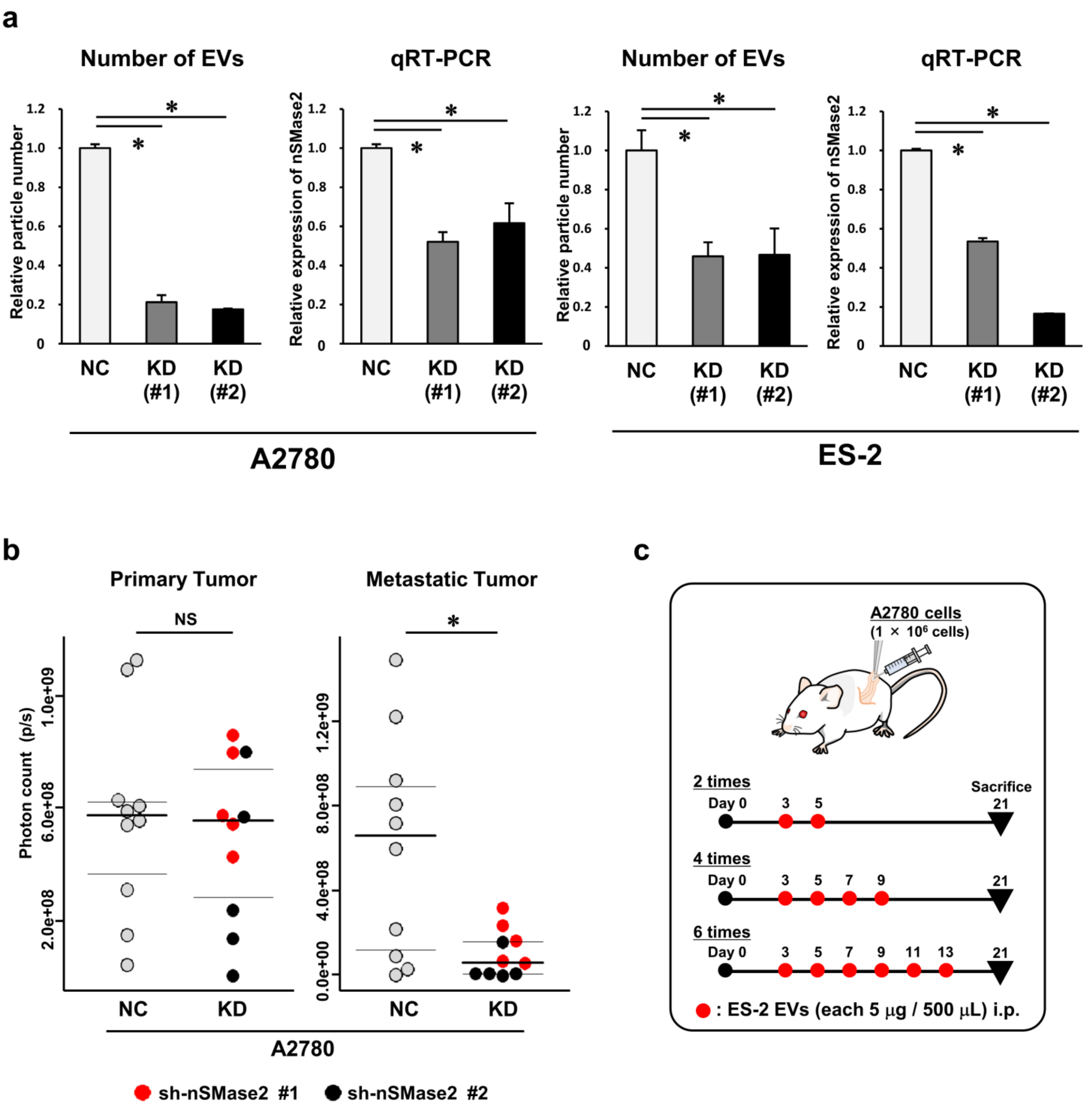

Supplementary Figure 3. ***In vivo* experiments determine dose-dependency of the EVs for peritoneal dissemination of cancer cells** (a) Characterization of nSMase2 knockdown (KD) cells. A2780 cells and ES-2 cells were transfected with the two different sequence of nSMase2 shRNA vector (#1 and #2) or a negative control (NC) vector, and stable KD and NC cell lines were established. The number of EVs secreted from the same number of cells using Nanosight is shown in each left panel. The expression level of nSMase2 in each cell determined by qRT-PCR, and actin was used as an internal control. The data are shown in each right panel. Error bars represent s.d., Dunnett's test (**p* < 0.05). (b) Distribution of photon count in the dissected primary tumors (left) and the peritoneal metastatic tumors (right) using nSMase2 knockdown (KD) cells and negative control (NC) cells. The orthotopic mouse model was established by injection with A2780 cells, and the cells were transfected with the two different sequence of nSMase2 shRNA vector (KD; #1 and #2) or control vector (NC). The mice were euthanized at day 30. KD; knockdown of nSMase2, NC; negative control. Student's t-test (**p* < 0.05 and NS = no significance). (c) Schematic protocol for investigating the different EV doses in peritoneal dissemination. Orthotopic mouse models were established with A2780 cells. ES-2 EVs were injected i.p. from day 3 and every other day thereafter. The names of the groups indicate how many times the EVs were injected. The mice were euthanized on day 21. The results of this experiment are shown in Figure 2e.

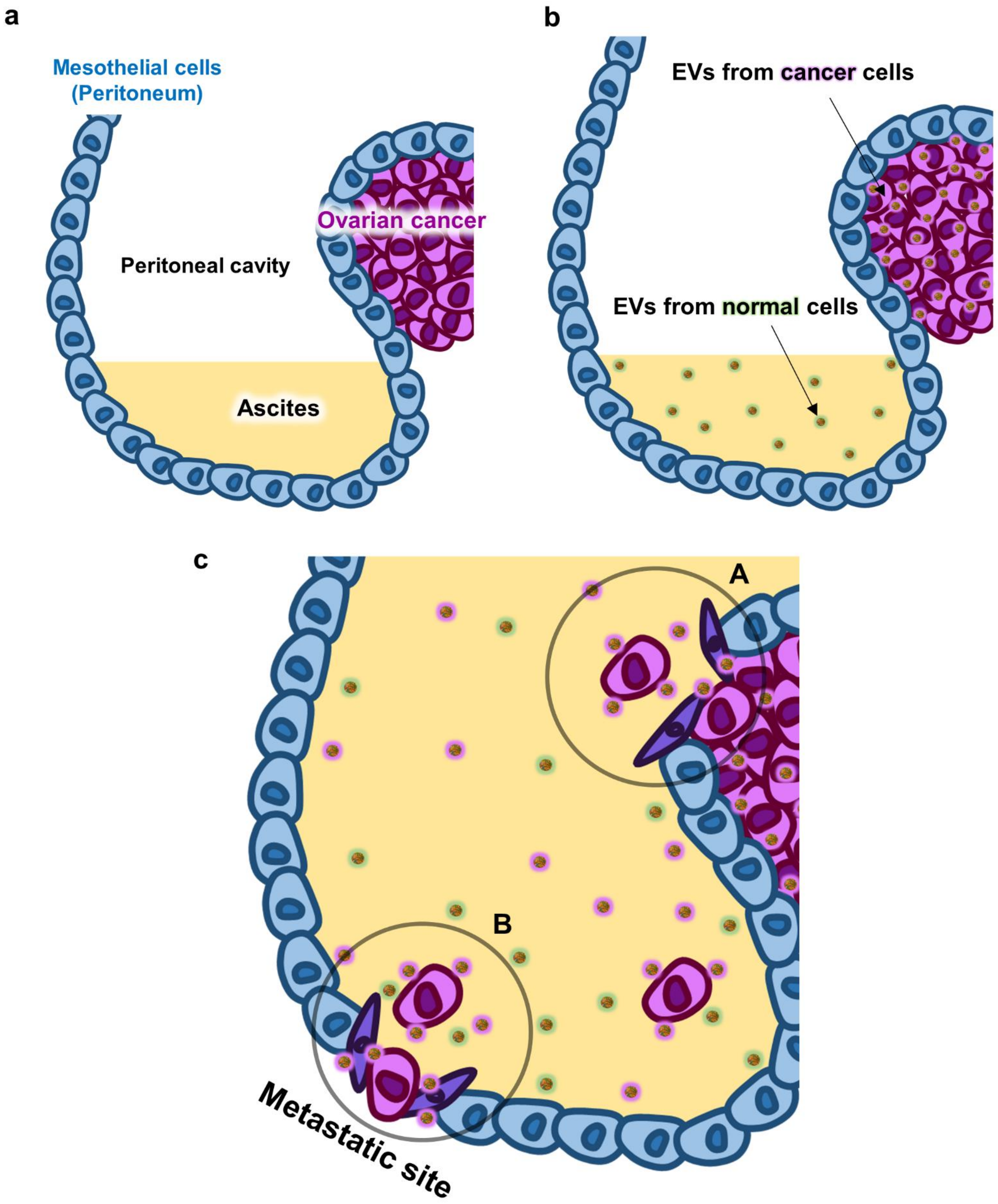

Supplementary Figure 4. Schematic diagram for cancer progression in the peritoneal cavity (a) The description of the names in each part of the diagram. The blue cells indicate mesothelial cells, which are the major components of the peritoneum and are single-layered. The purple cells indicate cancer cells, and the yellow area indicates ascites. (b) The description indicating the EVs from cancer cells (purple) and normal cells (green). The EVs can be found in ascites. (c) A hypothetical diagram of peritoneal dissemination mediated by the EVs. In many cases, ascites accumulated in the peritoneal cavity. The EVs from cancer cells affect the mesothelial cells when the cell invade the peritoneal cavity from the primary tumor. (A) Blue mesothelial cells convert to purple abnormal cells. In theory, the effect would be favorable to progression of cancer cells. Then, these changes promote cancer cell attachment to the peritoneal wall, which will become metastatic sites (B).

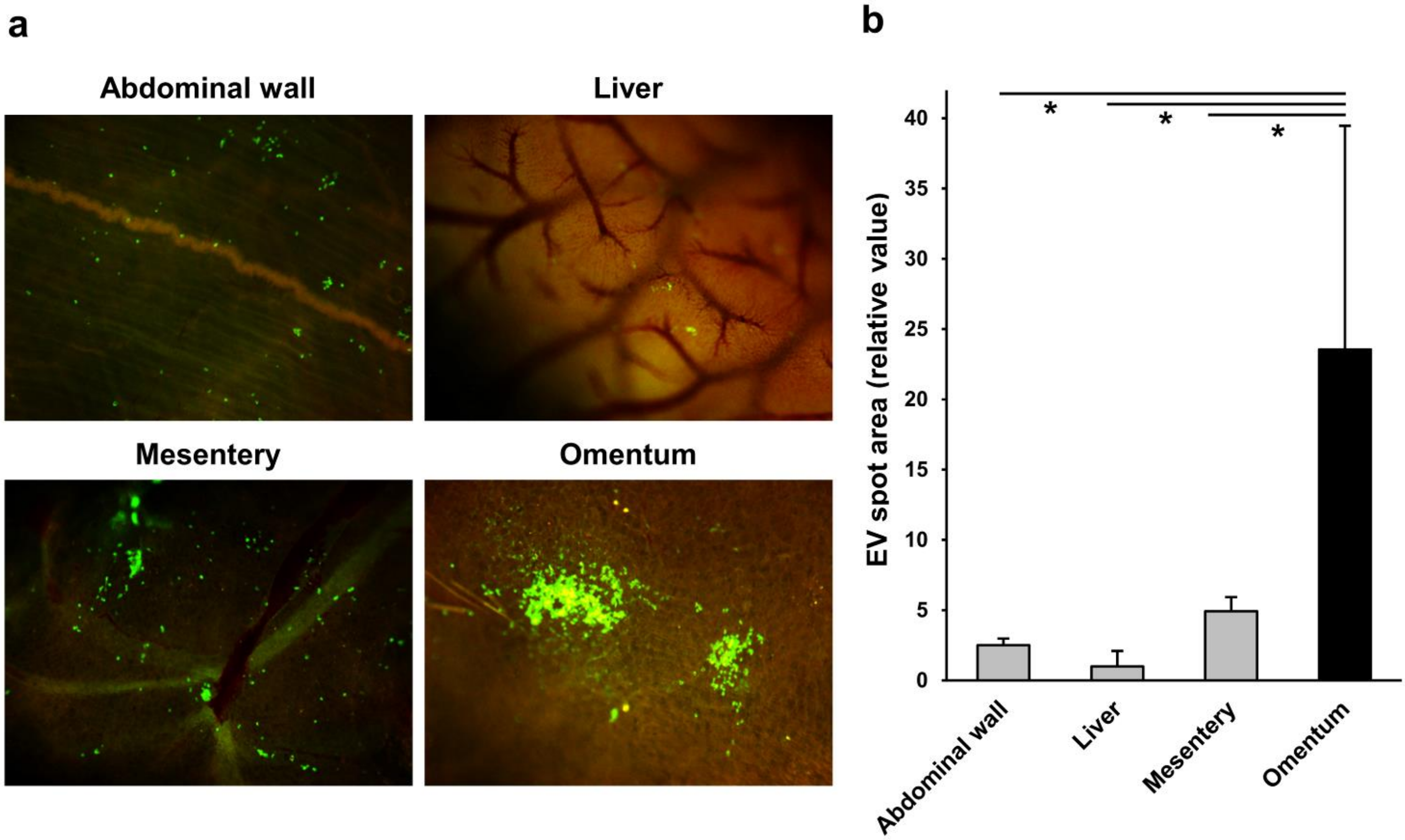

Supplementary Figure 5. EV attachment preferences for intra-abdominal organs (a) The representative stereomicroscopic images of EV accumulation. PKH67 green-labeled ES-2 EVs were injected i.p. into mice, and the abdominal wall, liver, mesentery and omentum were dissected after 12 h. The tissues were promptly observed by stereomicroscopy. n = 3 (b) Quantification of EV accumulation. To quantify EV accumulation in 4 types of tissue, the image analysis application for the BZ-X700 microscope (Keyence, Japan) was used. The areas with green fluorescence were measured. The value of liver was set to 1.0. Error bars represent s.d., Student's t-test (*$p < 0.01$).

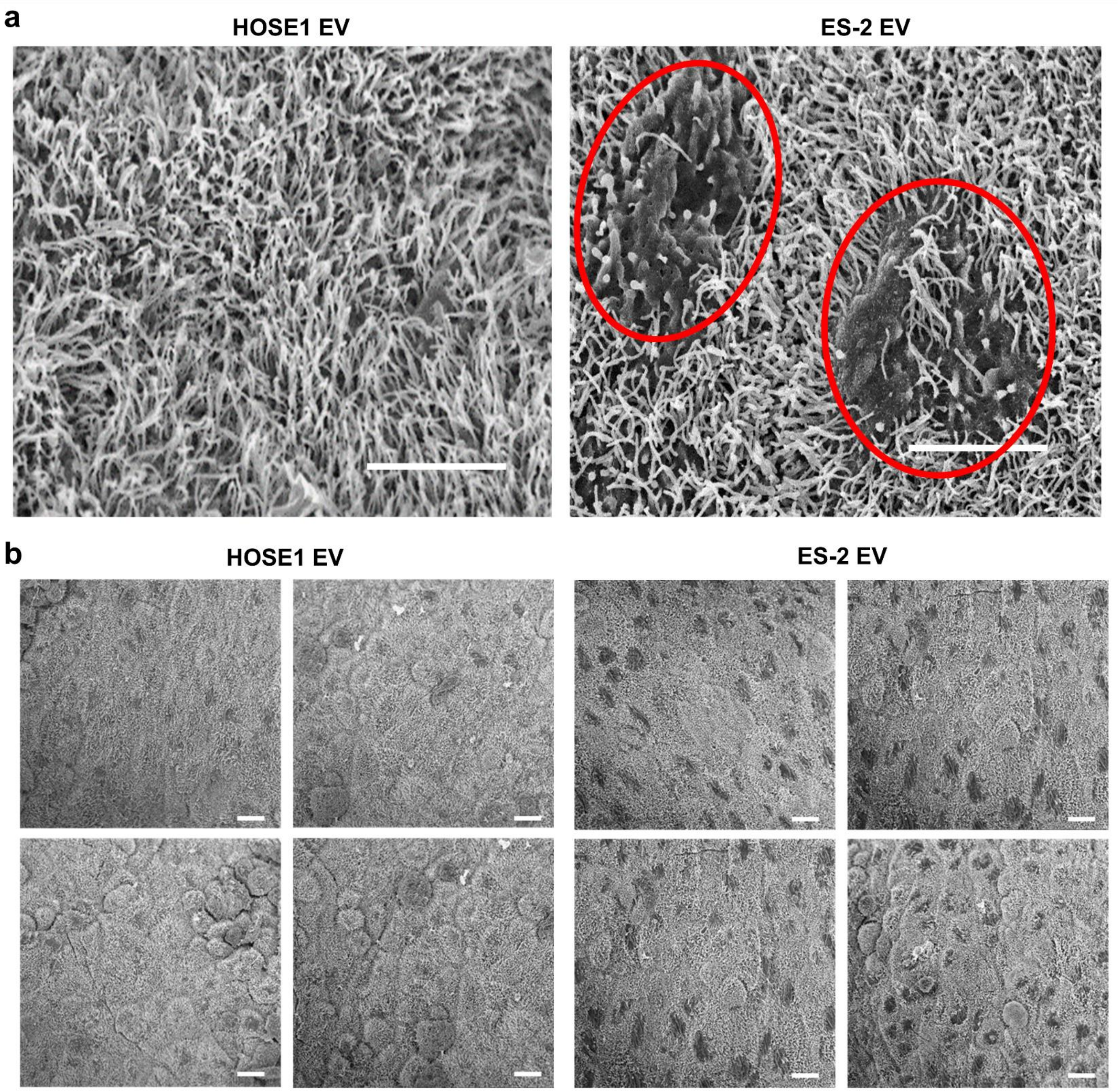

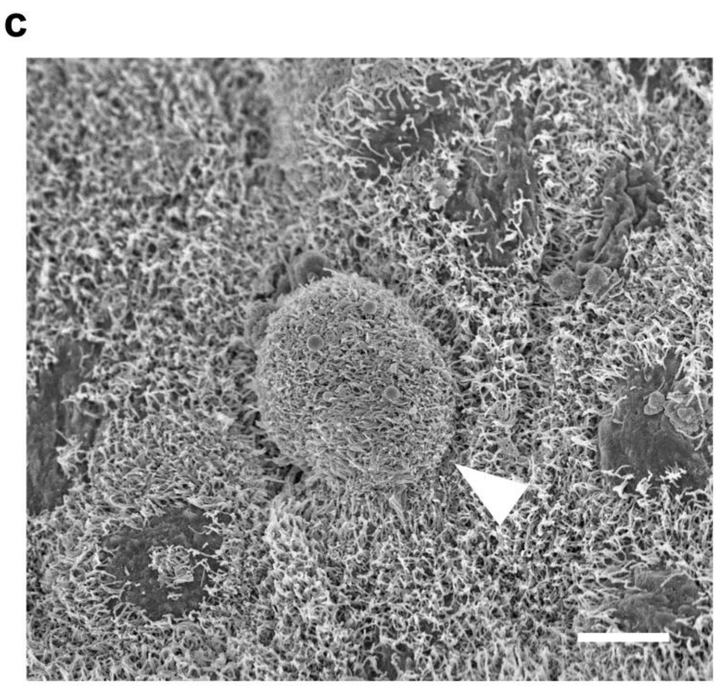

Supplementary Figure 6. Scanning electron microscopic (SEM) images of the peritoneum of mice treated with EVs (a) Representative SEM images of abdominal walls treated with EVs. Red circles indicate the defective sites without microvilli. Scale bars, 10 μm. (b) Multiple SEM images of abdominal walls treated with EVs. Scale bars, 5 μm. (c) The representative images of exfoliation of the mesothelial cells (wihte arrowheads). The spherical object in the center of this image could indicate a mesothelial cell just detaching from the abdominal membrane. Scale bars, 10 μm.

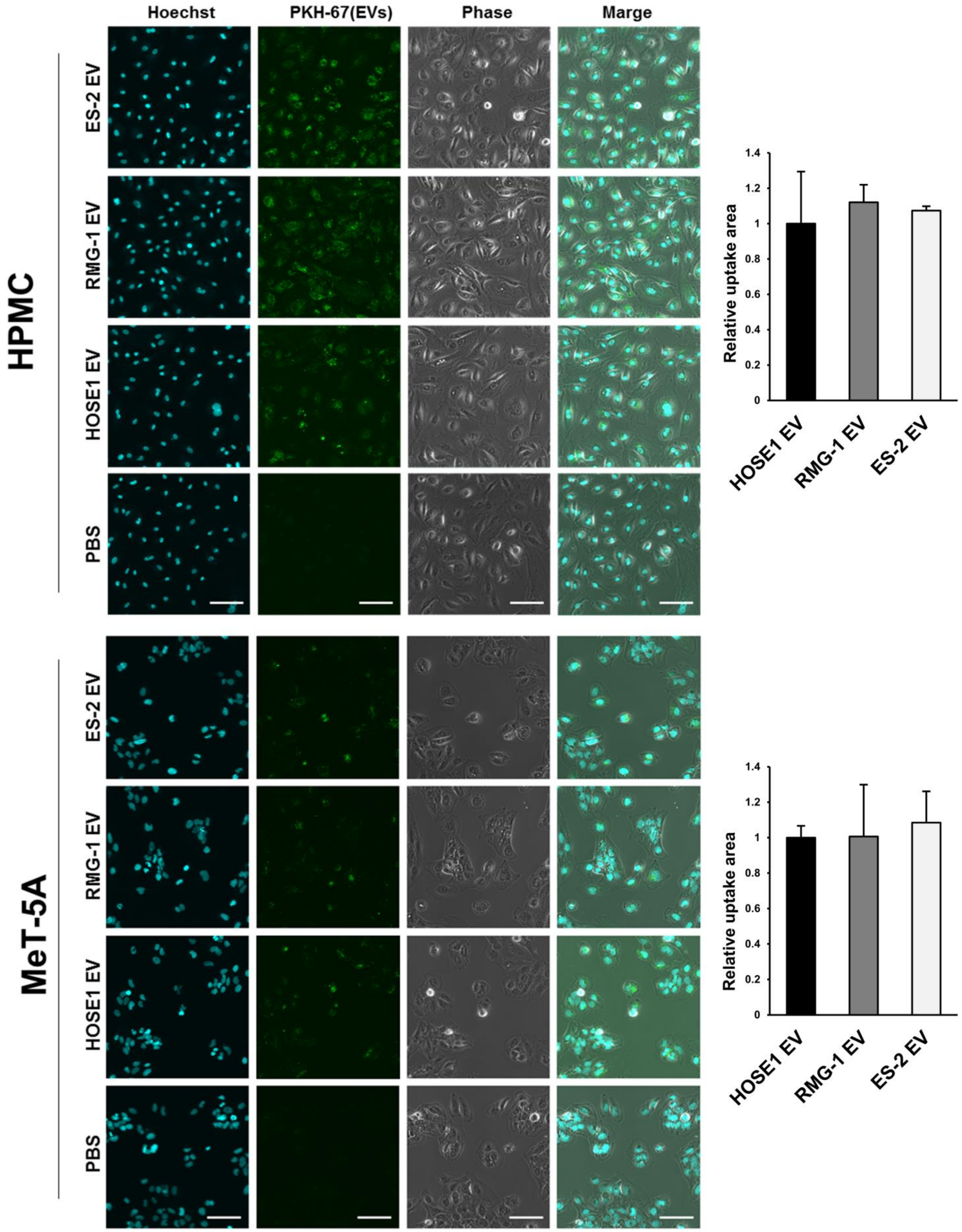

Supplementary Figure 7. Uptake of EVs in mesothelial cells *in vitro* Representative confocal microscopic images. EVs derived from ES-2 cells, RMG-1 cells and HOSE1 cells were labeled by PKH67 green and added to two types of mesothelial cells (MeT-5A and HPMC). Control indicates PBS with PKH67 green. Scale bar, 100 μm. The uptake of EVs by mesothelial cells (MeT5A and HPMC) was quantified using the image analysis application for the BZ-X700 (Keyence, Japan). EV areas were normalized with the number of nuclei in 5 randomly selected fields and expressed as relative values.

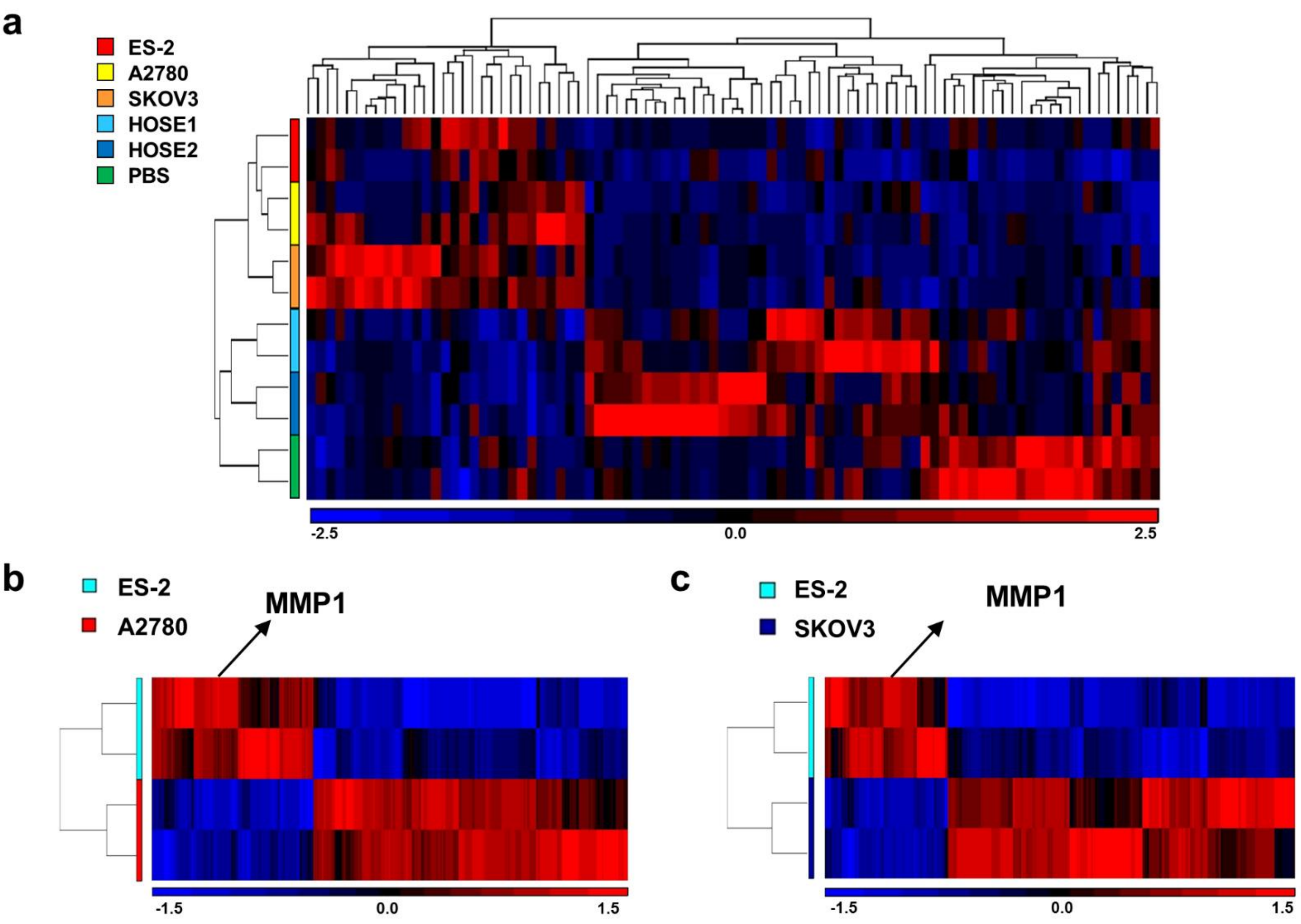

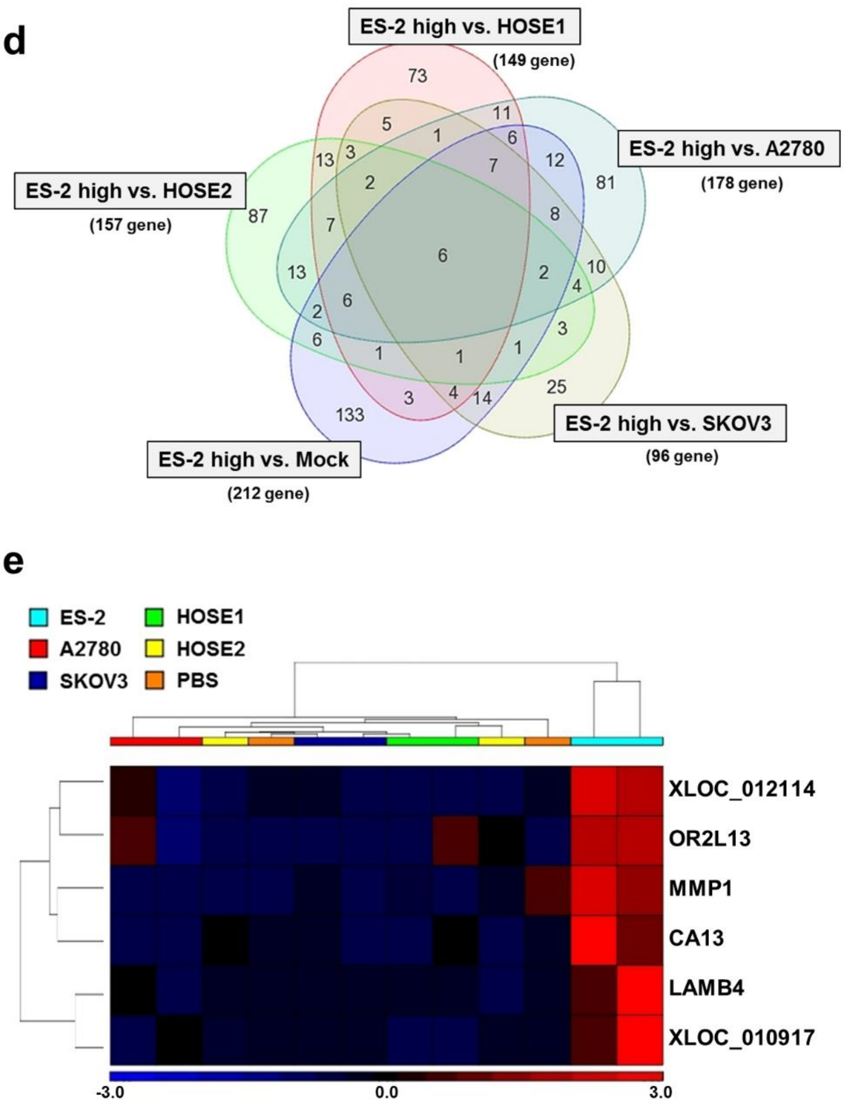

Supplementary Figure 8. Gene expression analysis in the mesothelial cells treated with different EVs (a) A heat map showing 89 differentially expressed genes in MeT-5A cells treated with the various EVs and PBS. Three cancer cell lines (ES-2, A2780 and SKOV3) vs. HOSE1&2 and Mock. (time point: 48 h, change > 2 fold and p < 0.05). (b) A heat map showing 524 differentially expressed genes (time point: 48 h, ES-2 EVs vs A2780 EVs, change > 2-fold and p < 0.05) in MeT-5A cells treated with the EVs. (c) A heat map showing 367 differentially expressed genes (time point: 48 h, ES-2 EVs vs SKOV3 EVs, change > 2-fold and p < 0.05) in MeT-5A cells treated with the EVs. (d) Investigation of the specific gene in the mesothelial cells treated with ES-2 EV as shown in Venn diagram (change > 2-fold and *p* < 0.05). (e) A heat map of 6 specifically up-regulated gene (from Venn diagram) in the mesothelial cells treated with ES-2 EV.

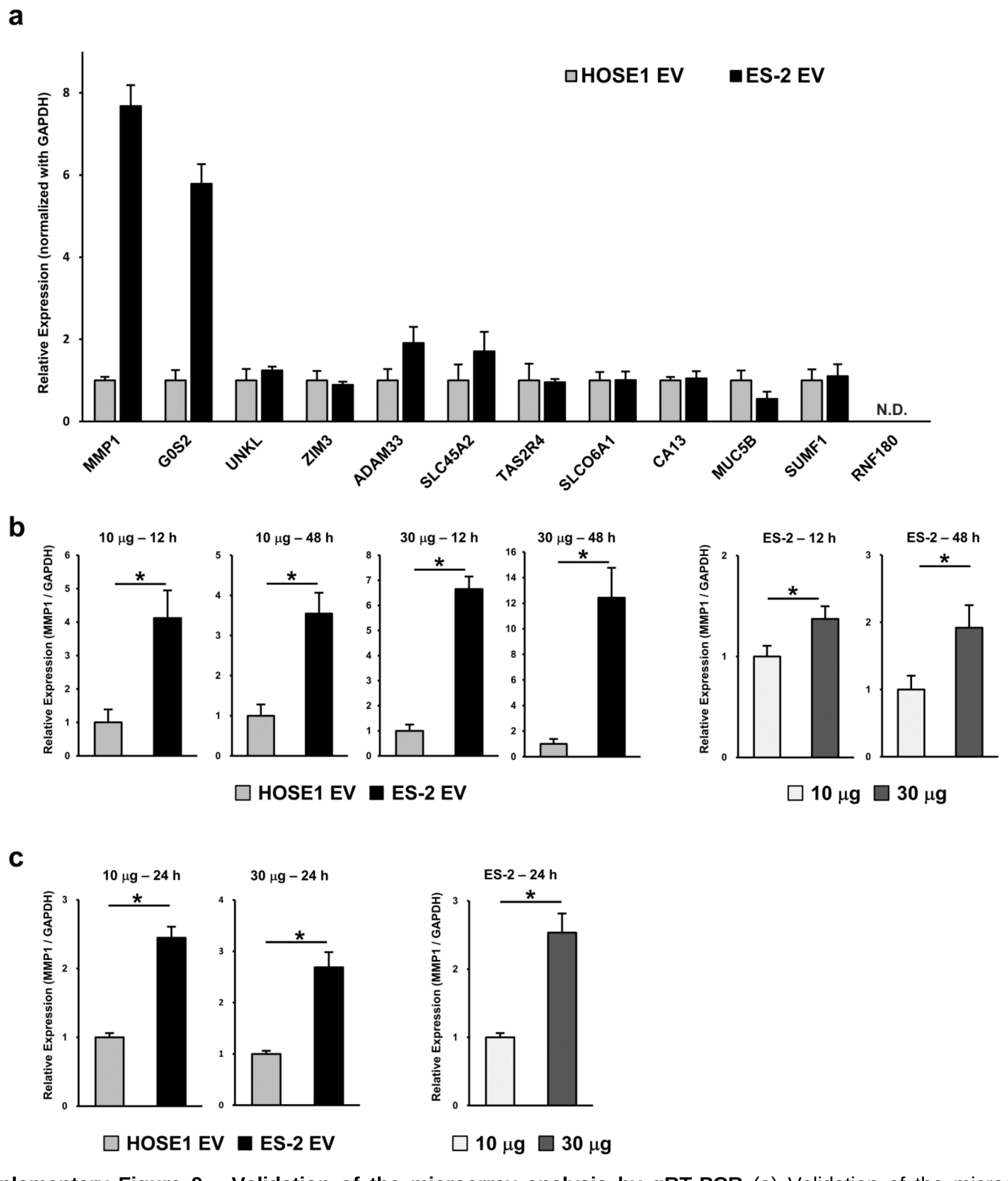

Supplementary Figure 9. Validation of the microarray analysis by qRT-PCR (a) Validation of the microarray analysis in independent experiments. Expression level of the top 12 mRNAs in mesothelial cells (MeT-5A) at 48 h after the addition of ES-2 EVs and HOSE1 EVs. The expression was measured by qRT-PCR, and GAPDH was used as an internal control. (b) Validation of the microarray analysis focusing on MMP1 mRNA. Expression level of MMP1 mRNA in MeT-5A cells after the addition of ES-2 EVs and HOSE1 EVs as described in Fig. 2a (2 time points and 2 two different amounts). Expression was measured by qRT-PCR, and GAPDH was used as an internal control. Error bars represent s.d., Student's t-test (*p < 0.01). (c) Expression level of MMP1 mRNA in HPMCs after the addition of ES-2 EVs and HOSE1 EVs (1 time point and 2 two different amounts). Expression was measured by qRT-PCR, and GAPDH was used as an internal control. Error bars represent s.d., Student's t-test (*p < 0.01).

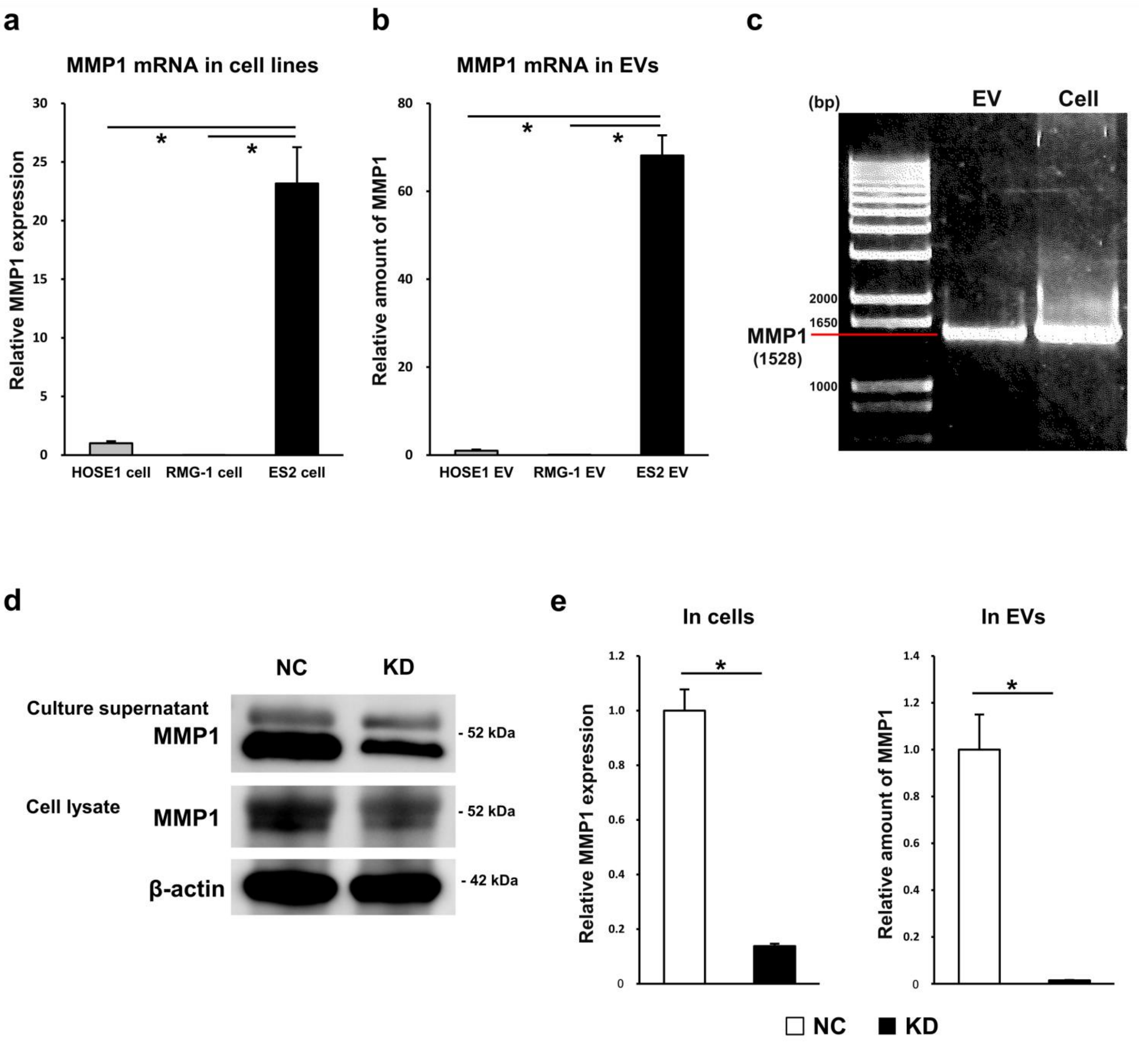

Supplementary Figure 10. Investigation of MMP1 mRNA in EVs (a) The expression level of MMP1 in cell lines. The expression of MMP1 in ES-2 cells, RMG-1 cells and HOSE1 cells was measured by qRT-PCR, and GAPDH was used as an internal control. Error bars represent s.d., Student's t-test (*$p < 0.01$). (b) The amount of MMP1 mRNA in EVs. The amount of MMP1 mRNA in EVs from ES-2 cells, RMG-1 cells and HOSE1 cells was measured by qRT-PCR. The same amount of EVs (30 μg) was used for this analysis. Error bars represent s.d., Student's t-test (*$p < 0.01$). (c) Investigation of the size of the MMP1 mRNA in ES-2 EVs. Total RNA was extracted from ES-2 cells and ES-2 EVs, and RT-PCR was then performed. The product size of MMP1 mRNA was determined using custom primers (Supplementary Table 1; MMP1 full-length primer) and was estimated to be 1528 bp. (d) Characterization of stable MMP1 KD cell lines by immunoblot analysis. Proteins were obtained from the cell culture supernatant and cell lysate from stable MMP1 KD cells and negative control (NC) cells. MMP1 and b-actin were investigated. (e) Characterization of stable MMP1 KD cell lines by qRT-PCR. Total RNA was extracted from ES-2 MMP1 KD and NC cells and the EVs, and qRT-PCR was then performed. The expression level of MMP1 in the cell lines was normalized to GAPDH (left graph). The same amount of EVs (30 μg) was used for this analysis (right graph). Error bars represent s.d., Student's t-test (*$p < 0.01$).

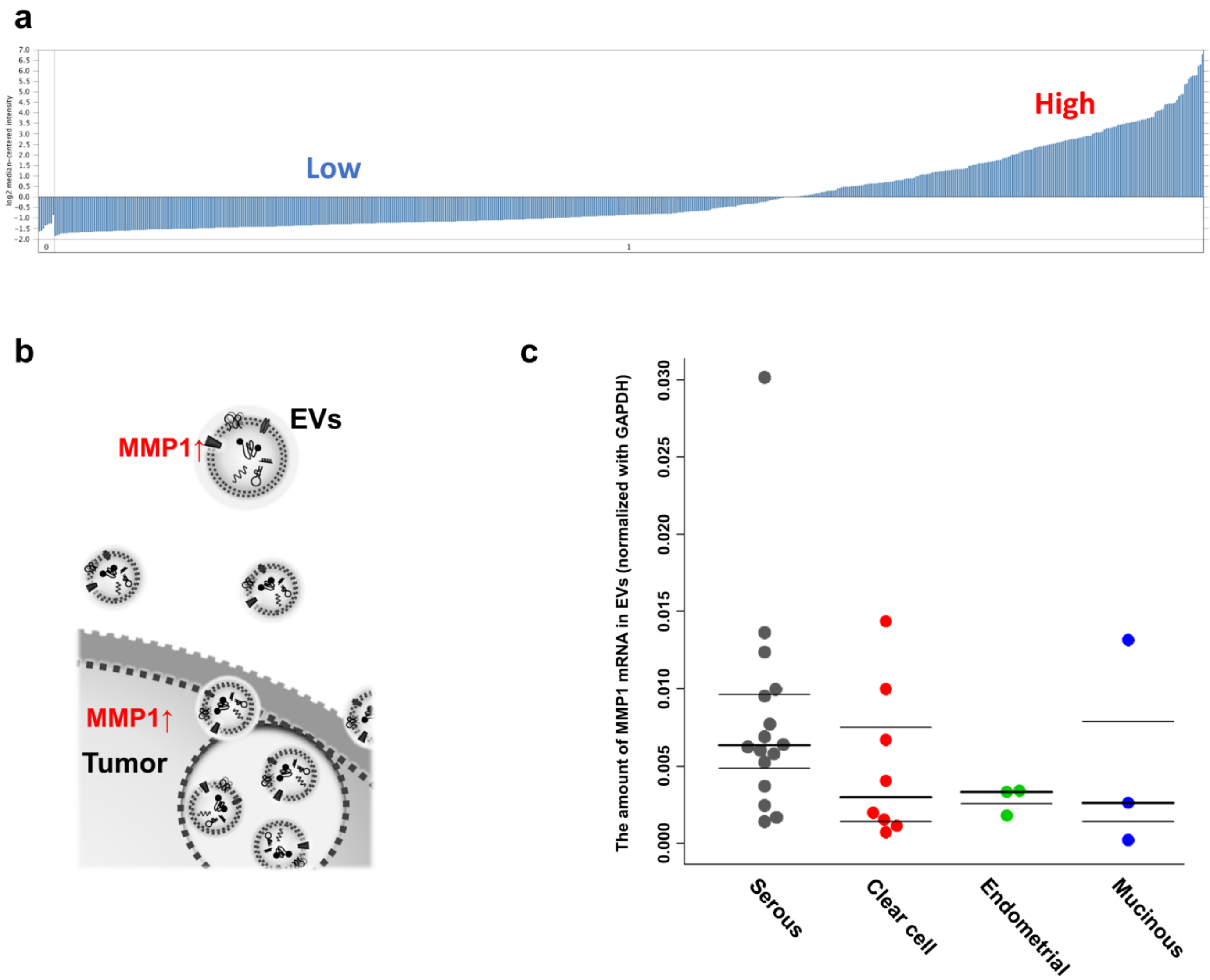

Supplementary Figure 11. Clinical relevance of MMP1 in ovarian cancer (a) The expression level of MMP1 in ovarian cancer tissues. The Oncomine (www.oncomine.org) database was used to evaluate the clinical relevance of MMP1 in ovarian cancer. Using the TCGA database, 594 samples were analyzed. (b) Schematic diagram for the distribution of MMP1 mRNA. Cells or tissues with high expression of MMP1 can release EVs containing many MMP1 mRNAs into the culture supernatant or body fluids, such as ascites. (c) Distribution of MMP1 mRNA in patients' ascites-derived EVs. The graph indicates the differences among 4 histopathological subtypes. n = 16; Serous, 8; Clear cell, 3; Endometrial and 3; Mucinous. The amount of MMP1 mRNA in the EVs was normalized to GAPDH.

Uncropped blots for Figure 5f

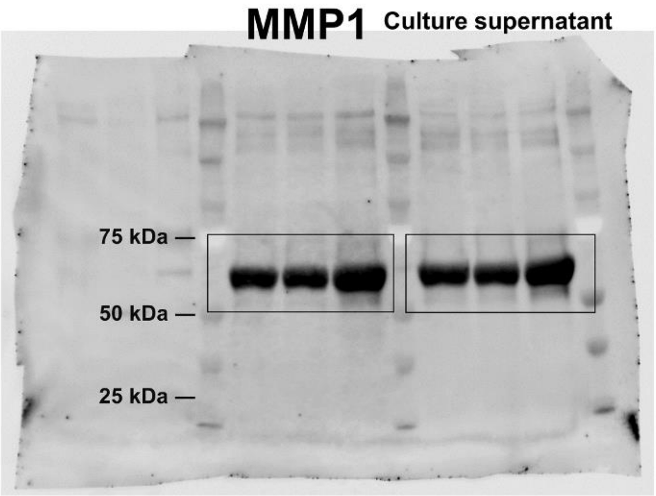

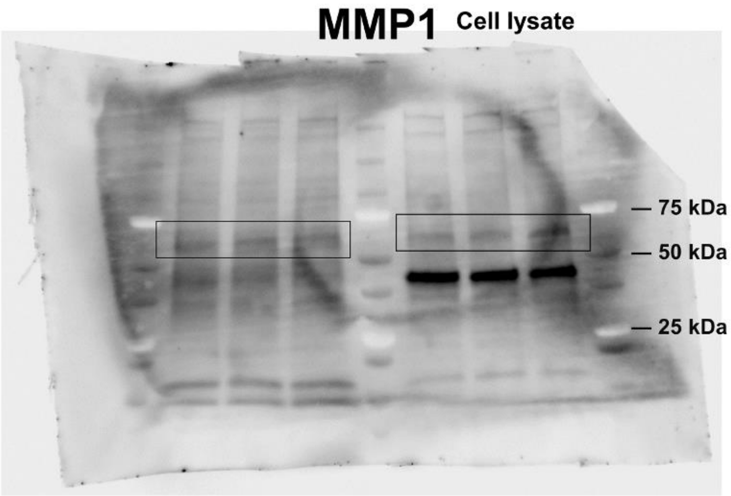

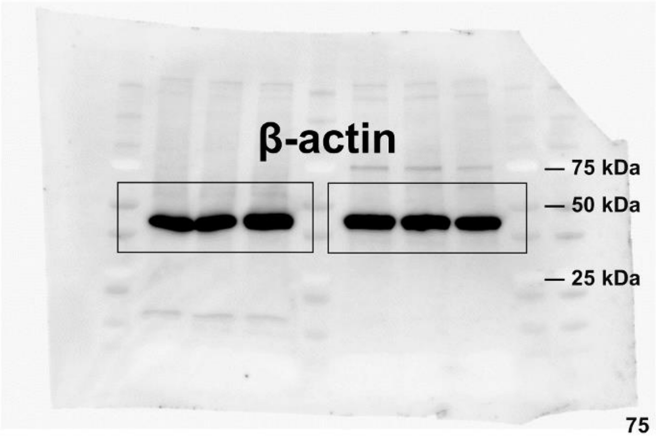

Uncropped blots for Supplementary Figure. 1c

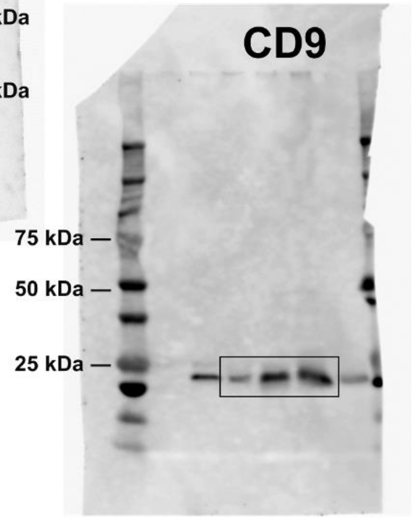

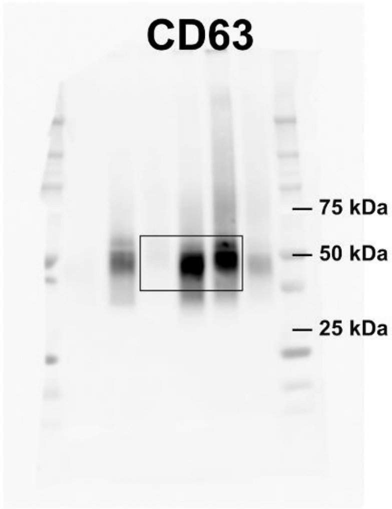

Uncropped blots for Supplementary Figure. 8

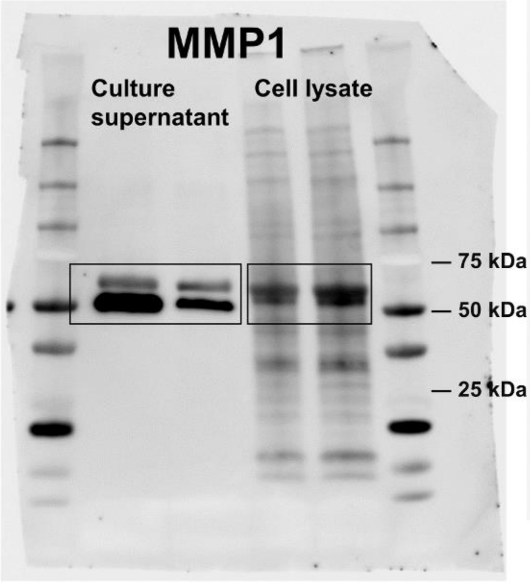

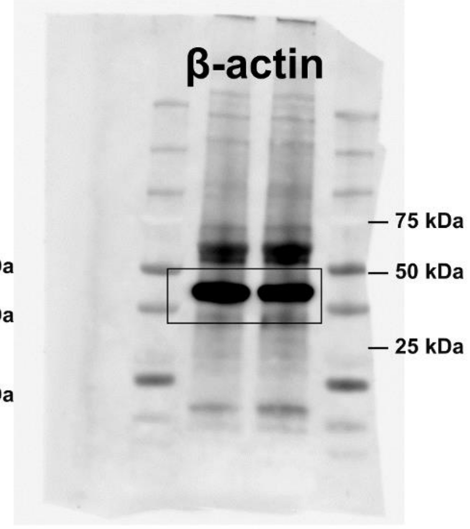

Supplementary Figure. 12 Uncropped immunoblots

a

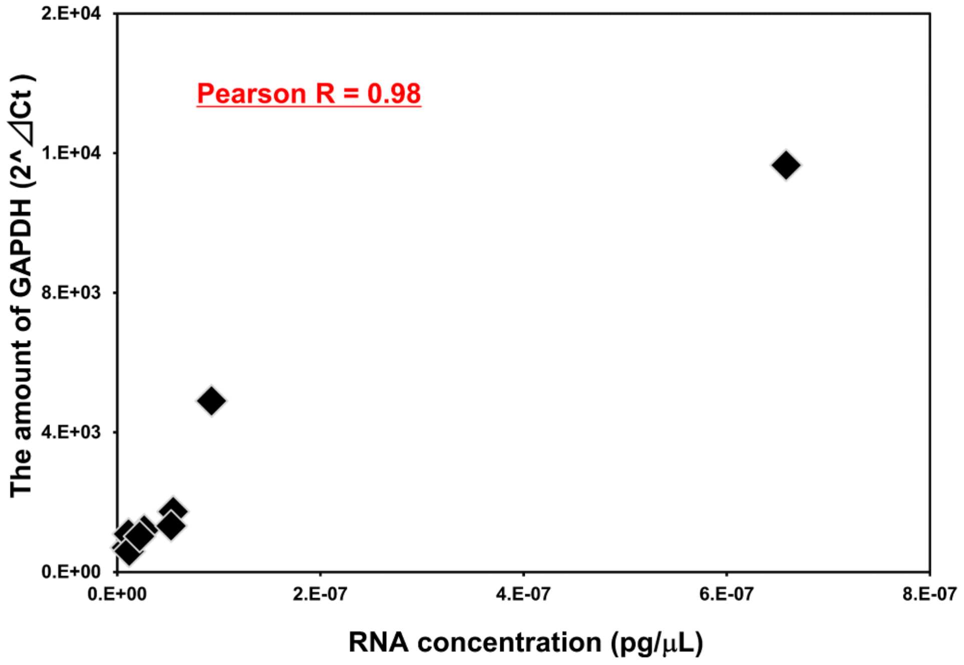

b

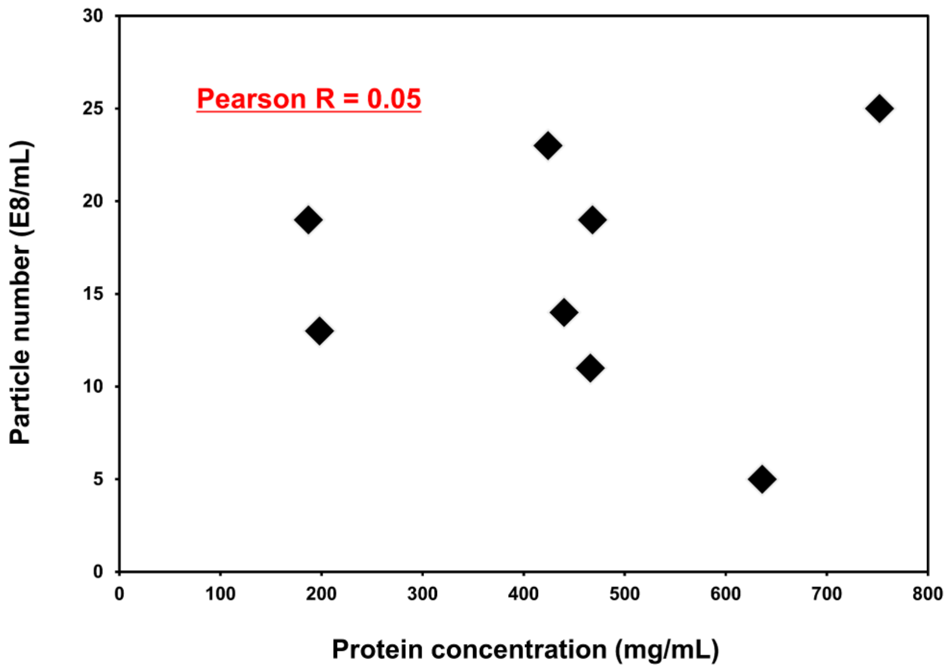

Supplementary Figure 13. Correlation analysis of index values for normalizing the EVs (a) Correlation between the amount of total RNA and GAPDH in EVs from patients' ascites. Ten patient ascites samples were randomly selected. The EVs were isolated from 5 mL of ascites, and total RNA was extracted. The amount of total RNA was measured by a bioanalyzer (Agilent) and that of GAPDH by qRT-PCR. Pearson's R value is shown in the graph. (b) Correlation between the amount of proteins and the number of particles in EVs from patients' ascites. Eight patient ascites samples were randomly selected. The EVs were isolated from 5 mL of ascites, and the amount of proteins and the number of particles were measured. Pearson's R value is shown in the graph.

Supplementary Table 1. Significant enrichment of gene pathways in EV-treated MeT-5A cells

Cell Line	NAME	SIZE	NES	NOM p-val
ES-2	MRNA_PROCESSING_GO_0006397	46	1.54	>0.001
ES-2	POSITIVE_REGULATION_OF_CASPASE_ACTIVITY	21	1.52	>0.001
ES-2	VITAMIN_METABOLIC_PROCESS	15	1.48	>0.001
ES-2	CASPASE_ACTIVATION	19	1.45	>0.001
ES-2	BRAIN_DEVELOPMENT	40	1.41	>0.001
ES-2	PROTEOLYSIS	136	1.36	>0.001
ES-2	ELECTRON_TRANSPORT_GO_0006118	42	1.34	>0.001
ES-2	PROTEIN_LOCALIZATION	159	1.29	0.021
ES-2	I_KAPPAB_KINASE_NF_KAPPAB_CASCADE	85	1.32	0.031
ES-2	MACROMOLECULE_LOCALIZATION	172	1.30	0.044
ES-2	MRNA_METABOLIC_PROCESS	55	1.51	0.049
A2780	POSITIVE_REGULATION_OF_HYDROLASE_ACTIVITY	38	1.61	>0.001
A2780	PROTEIN_AUTOPROCESSING	23	1.44	>0.001
A2780	PROTEIN_AMINO_ACID_AUTOPHOSPHORYLATION	22	1.44	>0.001
A2780	POSITIVE_REGULATION_OF_CASPASE_ACTIVITY	21	1.50	0.023
A2780	MACROMOLECULE_LOCALIZATION	172	1.24	0.029
A2780	EMBRYONIC_DEVELOPMENT	40	1.38	0.043
SKOV3	POSITIVE_REGULATION_OF_CELLULAR_COMPONENT_ORGANIZATION_AND_BIOGENESIS	30	1.34	>0.001
SKOV3	MRNA_PROCESSING_GO_0006397	46	1.46	0.032
SKOV3	OXYGEN_AND_REACTIVE_OXYGEN_SPECIES_METABOLIC_PROCESS	17	1.44	0.034
SKOV3	RESPONSE_TO_LIGHT_STIMULUS	36	1.35	0.044
3 cell lines	GENERATION_OF_NEURONS	63	1.44	0.013
3 cell lines	ELECTRON_TRANSPORT_GO_0006118	42	1.54	0.014
3 cell lines	NEUROGENESIS	71	1.47	0.021
3 cell lines	BRAIN_DEVELOPMENT	40	1.57	0.022
3 cell lines	PROTEIN_PROCESSING	36	1.40	0.028
3 cell lines	PROTEIN_AMINO_ACID_AUTOPHOSPHORYLATION	22	1.39	0.034
3 cell lines	GENERATION_OF_PRECURSOR_METABOLITES_AND_ENERGY	103	1.35	0.034
3 cell lines	PROTEIN_AUTOPROCESSING	23	1.39	0.034
3 cell lines	POSITIVE_REGULATION_OF_CYTOKINE_BIOSYNTHETIC_PROCESS	19	1.54	0.034
3 cell lines	NEURON_DIFFERENTIATION	58	1.36	0.038

The list from the gene set enrichment analysis (GSEA) of the mesothelial cells (Met-5A) treated with 3 cancer EVs versus those with HOSE1 EVs. Four types of comparison were performed, and significantly enriched pathways are listed.

Supplementary Table 2. Characteristics of patients

**Non-caner**	**N = 19**
ovarian benign diseases	7
uterine benign diseases	5
LPM	
mucinous	6
endometrioid	1

**Cancer**	**N = 41**
Histopathological types	
Serous	27
Clear cell	8
Mucinous	3
Endmetrioid	3
Stage	
I	11
II	1
III	21
IV	8
Therapy	
PDS	30
IDS	14

Ascites were collected from 60 patients, including 41 cancer patients and 19 non-cancerous patients. Three patients overlapped in the PDS and IDS groups because pair-samples from these patients were collected before neoadjuvant chemotherapy and during surgery. LPM; low potential malignancy. PDS; primary debulking surgery. IDS; intermediate debulking surgery.

Supplementary Table 3. Primer sequences for qRT-PCR analyses

Gene	Forward primer (5'-Sequence-3')	Reverse primer (5'-Sequence-3')
MMP1	aggtctctgagggtcaagca	ctggttgaaaagcatgagca
G0S2	taccacaagcatccaccaaa	tccttcctccctagtgcaaa
RNF180-F1	ggccaaagacaatccttcaa	atgcttttctgcagcttggt
UNKL-F1	actgagaagccgacccacta	agtacacgtcggggctgtag
ZIM3-F1	ctggccccaggatacataga	ttactcgcccttggtagtgg
ADAM33-F1	ctggccccaggatacataga	ttactcgcccttggtagtgg
SLC45A2-F1	agaagggcctccactaccat	gtgagcaccaatgcagagaa
TAS2R4-F1	aaaatgccactggtttctgg	ggaccagggtagcaactgaa
SLCO6A1-F1	tgacaaactgcgttctctgg	aacaacgtcctgtgtgtcca
CA13-F1	acaggttacggcaggttcac	tgaacaacatggagctctgc
MUC5B-F1	tccactatgagtgcgagtgc	aagcgtgcatggatctctct
SUMF1-F1	gcacctgcgaggagagttac	tgtctccagttagcgccttt
MMP1 full-length	gatattggagcagcaagagg	caccttctttggactcacac
GAPDH	gcaccgtcaaggctgagaac	tggtgaagacgccagtgga
